# Implementing Universal Whole‐Person Screening in High Schools: Lessons From a School–Health System Partnership

**DOI:** 10.1111/josh.70260

**Published:** 2026-10-18

**Authors:** Dhruv Khurana, Geoffrey Leung, Bijan Sasaninia, Janine Moore, Tracy Piper, Anthony Firek

**Affiliations:** ^1^ Department of Health Policy & Management University of California, Los Angeles, Fielding School of Public Health USA; ^2^ Comparative Effectiveness and Clinical Outcomes Research Center (CECORC) Riverside University Health System USA; ^3^ Department of Population Health Sciences Weill Cornell Medicine USA; ^4^ Riverside University Health System—Medical Center USA; ^5^ University of California, Riverside School of Medicine USA; ^6^ Riverside University Health System—Behavioral Health USA; ^7^ Desert Sunset, LLC USA; ^8^ Department of Internal Medicine, Diabetes and Endocrinology Riverside University Health System USA

**Keywords:** adolescent health, behavioral health, implementation, mental health screening, school health services, school–county partnerships, social determinants of health, whole person health

## Abstract

**Background:**

Most school districts lack infrastructure to systematically identify whole‐person needs and connect students to coordinated services. We developed and implemented a whole‐person screening tool within a county–school partnership model.

**Methods:**

Between 2020 and 2024, Riverside University Health System—Behavioral Health partnered with a Southern California unified school district to implement the Whole Person Health Score‐Adolescent Version (WPHS‐AV) for 11,786 high school students. The partnership included universal screening, governance structures, tiered triage workflows, and school‐embedded behavioral health teams.

**Results:**

The WPHS‐AV demonstrated acceptable internal consistency (Cronbach's *α* = 0.64–0.65). Screening identified substantial moderate‐need students (86.3% had ≥ 1 Yellow flag) likely invisible under traditional crisis‐triggered referral systems, while high‐need classifications remained relatively uncommon (19.6% had ≥ 1 Red flag).

**Implications for School Health Policy, Practice, and Equity:**

Universal whole‐person screening identified 86% of high school students with moderate needs likely missed by crisis‐triggered systems. This district‐health system partnership demonstrates that systematic screening requires formal governance structures, tiered triage workflows, and school‐embedded clinical teams to connect identified students to appropriate supports.

**Conclusions:**

Universal whole‐person screening at scale requires partnership infrastructure, including formal governance structures, clearly defined workflows linking assessment to intervention, and iterative problem‐solving for consent barriers and referral bottlenecks.

## 1 Introduction

Behavioral health diagnoses among U.S. adolescents have increased [1, 2], with approximately half of lifetime mental disorders beginning by age 14, supporting the rationale for early identification in school‐aged populations [3].

Adolescence represents a critical developmental period characterized by executive function maturation, identity exploration, and reorganization of how individuals perceive and influence their internal and external environments [4, 5]. During the COVID‐19 pandemic, 37.1% of U.S. high school students reported poor mental health and 44.2% reported persistent sadness or hopelessness [6]. Pandemic‐era disruptions highlighted the breadth of factors affecting student wellbeing, including technology barriers, reduced peer interaction, household financial instability, and loss of adult mentorship [7, 8]. Despite high need, access remains limited: in national estimates, 60.3% of youth with major depression did not receive mental health treatment [9].

In California, chronic absence (defined as missing 10% or more of school days during the academic year for any reason) more than doubled during the pandemic, increasing from 12.1% (2018–2019) to 30% at the peak (2021–2022), signaling widening gaps between student needs and available supports [10]. California's Mental Health Services Act (Proposition 63) and the Mental Health Student Services Act (MHSSA) have created funding mechanisms supporting school–county partnerships focused on prevention and early identification [11].

Despite this policy momentum, many school districts still lack the infrastructure to systematically identify students' whole‐person needs and connect them to appropriate services. Several operational challenges help explain this gap [12]. First, anonymous student wellbeing surveys provide aggregate data useful for general programming but incapable of connecting specific students to services [13]. Second, resource constraints drive schools to focus primarily on students with overt behavioral or attendance crises (reactive “Tier 3” identification) [14], while students with internalizing symptoms or emerging needs remain overlooked [15] until problems escalate, missing the opportunity for lower‐intensity preventive intervention. Third, existing screening approaches rarely enable longitudinal tracking at the individual level [16], limiting districts' ability to assess whether interventions improve trajectories.

A range of models have sought to bridge health and education, including school‐based health centers [17], full‐service community schools [18], and whole‐school approaches [19]. While school‐based health centers (SBHCs) operate in about 3900 sites nationwide [20] and deliver on‐site clinical services and care coordination [21], most focus primarily on direct service provision within a single school site. By contrast, district‐wide screening models can link identified needs to predefined triage and referral pathways that route students to different levels of school‐based, social, and behavioral health support [22].

To address these gaps, we developed a district–health system partnership designed to enable student‐level identification of whole‐person needs paired with tiered linkage to appropriate supports. As part of this partnership, we adapted the validated Whole Person Health Score (WPHS) [23] for adolescents, creating the WPHS‐Adolescent Version (WPHS‐AV) to assess domains spanning physical, mental, social, and emotional well‐being. The program was implemented in collaboration with a large, unified school district in Southern California beginning in Fall 2020. It was developed in response to stakeholder‐identified concerns regarding student well‐being, attendance, and academic performance and the absence of a systematic approach to identify and address underlying needs.

We describe the partnership model and implementation workflow, including data governance and triage procedures; screening reach and characterization of identified needs; and tool feasibility, domain‐level need patterns, and internal consistency. We document barriers, adaptations, and lessons learned to support replication.

## 2 Methods

### 2.1 Study Design and Setting

We conducted an implementation evaluation of a district–health system partnership that operationalized universal whole‐person screening to a tiered response pathway delivered jointly by school and county behavioral health staff. The program was implemented in a large, unified school district in Southern California serving approximately 17,500 high school students annually, with a predominantly Hispanic/Latino population (65%) and significant socioeconomic diversity.

### 2.2 Partnership Model and Clinical Infrastructure

In 2019, the district partnered with Riverside University Health System Behavioral Health (RUHS‐BH) through TOPSS (Transforming Our Partnerships for Student Success), an MHSSA‐supported collaboration combining universal screening, tiered school‐based supports, and behavioral health linkage. Formal governance structures and FERPA/HIPAA‐compliant information‐sharing protocols supported implementation. District‐based staff provided school and social supports, while bicultural and bilingual TOPSS teams provided clinical assessment, case management, family engagement, and behavioral health services. Detailed governance and staffing procedures are provided in the Appendix under “Partnership Model and Governance” section.

### 2.3 Participants

The analytic sample included 11,786 unique students who completed the WPHS‐AV at least once during the study period spanning Fall 2020 through Fall 2024 (nine screening waves), representing 67.7% of eligible students. Among these, 11,016 (93.5%) had complete demographic information.

The eligible population consisted of approximately 17,500 high school students across participating district schools during the implementation period. Hispanic/Latino representation among students with complete demographic data (65.3%) was similar to the district‐wide proportion (65.5% in Fall 2021) [24]. However, student‐level demographic information for nonparticipants was not available in the analytic dataset, limiting our ability to formally compare participants and nonparticipants. We, therefore, interpret prevalence estimates as reflecting students reached by the screening program rather than the full eligible student population.

### 2.4 Instrumentation

#### 2.4.1 Whole *PERSON* Health Score‐Adolescent Version (WPHS‐AV) Development

The WPHS‐AV was adapted from the validated Whole *PERSON* Health Score (WPHS) adult tool for ages 12–17. A multidisciplinary team including behavioral health specialists, pediatricians, substance use experts, and educators reviewed the adult tool to identify items for modification. Specifically, the team focused on factors that influence adolescent development and academic success, are actionable through school‐based or community resources, can be tracked longitudinally to assess trajectory changes, and finally, reflect whole‐person health (not just mental health symptoms) [25, 26]. Development involved iterative review by expert panels, weekly feedback from students/parents in the partnering district, and content validity assessment to ensure cultural sensitivity and developmental appropriateness [27, 28, 29].

The adolescent adaptation retained the six‐domain *PERSON* framework but modified item content to reflect developmental and school‐context relevance. Key changes included adding adolescent‐relevant physical health indicators such as dental health; incorporating school and home safety within Emotional Health; emphasizing reliable adult connection, belonging, coping ability, and extracurricular engagement within Resource & Resilience; and adapting Ownership & Activation to include grades, absenteeism, conscientiousness, future planning, and self‐rating. The final 30‐item instrument assesses six *PERSON* domains: *P*hysical Health, *E*motional Health, *R*esource & Resilience, *S*ocioeconomics, *O*wnership & Activation, and *N*utrition & Lifestyle. A detailed list of items included in the assessment tool is provided in Table 1.

**TABLE 1:** WPHS‐AV domain structure and item dimensions (30 items across 6 domains).

Domain	Question
Physical health: This is a measure of current physical condition and takes into account any physical medical conditions	Blood pressure
	Body mass index
	Chronic condition load
	Functional activity
	Dental
Emotional health: This is a measure of emotional, mental, and spiritual well‐being	Depression
	Anxiety
	Social support
	Relaxation
	Safety at school
	Safety at home
Resource & resilience: This is a measure of one's ability to respond to challenges in a healthy way	Emergency room, Hospital visits
	Reliable adult
	Love, protected, and important
	Coping ability
	Extracurricular activities
Socioeconomics: This is a measure of one's financial status, parental education and access to resources	Finances (access to daily necessities)
	Transportation
	Parent/caregiver education
Ownership and activation: This is a measure for perception of one's own health and one's responsibility in learning, including setting goals, making decisions, and actively engaging in education	Self‐rating
	School absenteeism
	School grades
	Conscientiousness (effort, completing tasks)
	Future plan
Nutrition and lifestyle: This is a measure of one's health habits, including how well one exercises, eats, and sleeps	Diet
	Physical activity
	Sleep
	Smoking/vaping
	Alcohol, substance use

*Note:* Dimensions assessed within each WPHS‐AV domain. Item descriptions indicate the construct measured (e.g., “substance use,” “social support”); exact question wording is copyrighted and not displayed. Each domain generates letter scores (A–Z) mapped to Green/Yellow/Red triage zones.

After completion, item responses within each domain are scored and combined into a weighted domain score, with higher scores indicating greater identified need. Each weighted domain score is then mapped to a letter grade from A to Z and grouped into one of three risk‐severity zones: Green (A–F), Yellow (G–O), or Red (P–Z).

This traffic light system [30] of categorizing scores into three colors for triage and referrals was based on the success of the adult WPHS tool, which has been seamlessly integrated into the county's primary clinics [23].

### 2.5 Care Flow Plan and Triage Workflows (Figure 1)

**FIGURE 1:**
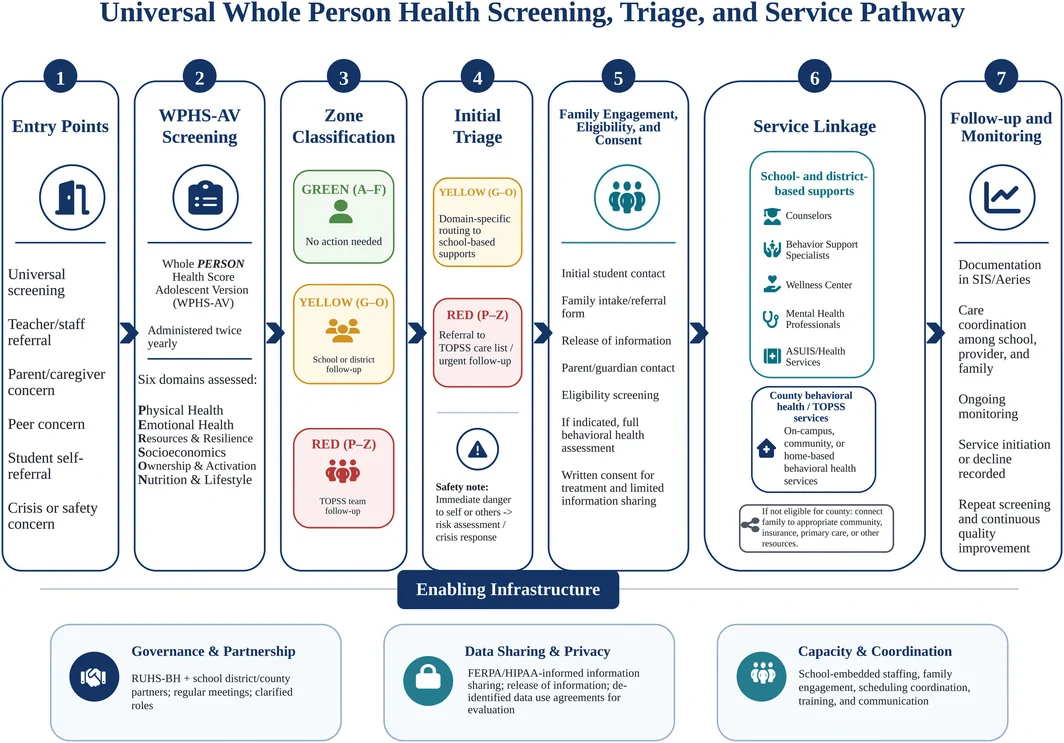
Universal whole person health screening, triage, and service pathway. Illustrative process model showing pathways from screening entry points through WPHS‐AV administration, zone classification, triage, family engagement and eligibility assessment, service linkage, and follow‐up/monitoring, with supporting governance, data‐sharing, and coordination infrastructure. ASUIS, Alcohol and Substance Use Intervention Specialists; TOPSS, Transforming Our Partnerships for Student Success.

When screening results are generated, the Care Flow Plan automatically maps zone classifications (Green/Yellow/Red) to defined actions by domain: for Green Zone classifications, students continue with universal supports (no additional intervention). Yellow‐zone classifications in Emotional Health, Resources & Resilience, Ownership & Activation, and Socioeconomics are routed to school‐based resources, including school counselors, school‐based mental health professionals (therapists/social workers), and the district Wellness Center for social needs support (food, housing, and transportation assistance). Yellow scores in the Nutrition & Lifestyle domain are routed to Alcohol and Substance Use Intervention Specialists (ASUIS) for targeted support. Red‐zone classifications across all domains trigger immediate referral to TOPSS teams for clinical follow‐up. When a student exhibits immediate safety concerns or presents signs of danger to self or others, school personnel initiate a structured risk assessment protocol, and decision‐making incorporates WPHS‐AV scores, family intake assessment data, and risk assessment findings.

Triage decision‐making authority rested with frontline staff (school counselors for Yellow‐zone students, TOPSS coordinators for Red‐zone students) rather than administrators, a refinement made after identifying administrative review as a bottleneck that delayed service connection.

### 2.6 Procedure

The district implemented WPHS‐AV beginning Fall 2020 via Qualtrics (secure web‐based platform) on a twice‐annual schedule (Fall and Spring semesters). Students accessed screening through supervised in‐class administration or completed it remotely via individualized links. Repeated assessments were linked across screening waves using district student identifiers, and demographic and student‐status characteristics were obtained from linked district administrative records. Outside the twice‐annual universal screening windows, students could also be identified through teacher referral, parent/caregiver concern, peer concern, or self‐referral; in these cases, the WPHS‐AV could be administered individually after obtaining case‐by‐case parental permission.

Parental consent for WPHS‐AV screening was obtained through an opt‐in checkbox embedded within the district's annual electronic school re‐registration module, an online process families complete each year to confirm enrollment information and review required school forms. The module contained approximately 20 consent statements covering various school programs and activities. For students referred outside universal windows (via teacher, peer, or self‐referral), parental permission was obtained case by case. Students accessing TOPSS clinical services required additional consent and release authorizations specifying information sharing between the health system and the school under FERPA/HIPAA safeguards. Consent materials were provided in multiple languages. The opt‐in approach prioritized explicit family permission but introduced implementation tradeoffs, including lower screening reach and possible inequities for families facing language, housing, work‐schedule, or administrative barriers.

#### 2.6.1 Service Connection Procedures

After a TOPSS referral, coordinators arranged a behavioral health assessment and Medi‐Cal eligibility screening. Eligible students whose families consented could receive TOPSS services; others were connected to alternative resources and remained eligible for district‐based supports. Information exchange and documentation followed applicable HIPAA and FERPA requirements. Detailed service‐connection procedures are provided in the Appendix under “Partnership Model and Governance” section.

### 2.7 Data Analysis

We assessed internal consistency using Cronbach's α for the total scale and individual domains. To evaluate whether missing demographic information biased risk‐zone assignment, we fit population‐averaged multinomial generalized estimating equations with exchangeable working correlation structures to account for within‐student correlation across domains. Descriptive analyses characterized zone classification prevalence by domain. All analyses were conducted in R v4.5.0.

### 2.8 Ethics Review

The evaluation protocol was reviewed by the RUHS Institutional Review Board. We obtained parental consent through opt‐in procedures, and student assent at the time of screening administration.

## 3 Results

### 3.1 Sample Characteristics

The analytic sample included 11,786 unique students (70,716 domain‐assessments across nine screening waves). Among these, 11,016 (93.5%) had complete demographic information. Screening attempts varied: 5132 (43.5%) completed the assessment once, 2927 (24.8%) twice, 1762 (14.9%) three times, 1068 (9.1%) four times, 540 (4.6%) five times, 231 (2.0%) six times, and 93 (0.8%) seven or more times. Primary analyses used first‐attempt scores to maximize comparability.

Student demographics (*n* = 11,016) are presented in Table 2: Most identified as Hispanic/Latino (7196; 65%), followed by White (2205; 20%) and Black/African American (751; 7%). Sex: female (5555; 50%), male (5439; 49%). Language: English only (6921; 63%), English learner (961; 9%). Special education services (1640; 15%), foster care experience (102; 1%), homelessness (192; 2%), free/reduced lunch eligible (4233; 38%), socioeconomically disadvantaged (5334; 48%).

**TABLE 2:** Sample demographics.

Characteristic	*N*	%
*N*	11,016	100
Race & ethnicity		
American Indian/Alaska Native	82	1
Asian	288	3
Black/African American	751	7
Hispanic/Latino	7196	65
Multiple races	460	4
Native Hawaiian/Pacific Islander	34	0
White	2205	20
Sex		
Female	5555	50
Male	5439	49
Non‐binary	22	0
Language fluency		
English Language Proficiency Assessments (ELPAC) Pending	1	< 0.1
English Learner EL	961	9
English only	6921	63
Initially Fluent English Proficient (IFEP)	523	5
Reclassified Fluent English Proficient (RFEP)	2610	24
Special education		
No	9376	85
Yes	1640	15
English learner		
No	10,058	91
Yes	958	9
Foster care		
No	10,914	99
Yes	102	1
Experiencing homelessness		
No	10,824	98
Yes	192	2
Access to free/reduced lunch		
No	6783	62
Yes	4233	38
Socioeconomically disadvantaged		
No	5682	52
Yes	5334	48

### 3.2 Assessment of Bias due to Missing Demographic Data

Completeness of demographics was not associated with Red versus Green assignment (OR = 1.11, 95% CI 0.92–1.36, *p* = 0.28). Students with complete demographics had modestly lower odds of Yellow versus Green (OR = 0.90, 95% CI 0.83–0.98, *p* = 0.020). These findings support the inclusion of all 11,786 students in subsequent analyses. Additional details are provided in the Appendix under (“Assessment of Potential Bias due to Missing Demographic Data” section).

### 3.3 Internal Consistency of Domain Scores

The six domain scores demonstrated modest internal consistency (Cronbach's *α* = 0.64 [95% CI 0.63–0.65]; standardized *α* = 0.65). Item‐drop analyses showed omitting Physical Health would increase *α* to 0.66, while dropping Ownership & Activation, Nutrition & Lifestyle, or Emotional Health would decrease *α* to 0.55–0.58. Corrected item‐total correlations ranged from 0.27 (Physical Health) to 0.61 (Ownership & Activation), and standardized inter‐item correlations ranged from 0.47 to 0.68. Item‐level missingness ranged from 0% to 19%, with the highest missingness for Physical Health.

These findings indicate acceptable internal consistency for a multidomain screener intended to identify distinct domains of student need rather than measure a single unidimensional construct. The observed reliability was lower than adult WPHS validation estimates [23]. This difference is expected given the developmental adaptation of item content, the school‐based adolescent population, and the intentionally heterogeneous construct coverage spanning physical health, emotional health, resilience, socioeconomic barriers, ownership/activation, and nutrition/lifestyle. Importantly, the adult WPHS has been used in safety‐net settings as a structured, multidomain, patient‐level screening framework, supporting the relevance of adapting the approach for adolescent school‐based implementation [31, 32].

### 3.4 Zone Classifications by Domain

Across six domains, most students had no Red flags (80.4% had zero Red flags, 15.6% had one, 3.2% had two, < 1% had three or more). Yellow flags were broadly distributed (13.7% had zero, 18.5% had one, 20.7% had two, 20.9% had three, 16.2% had four, 8.0% had five, 2.0% had all six).

#### 3.4.1 Domain‐Specific Classifications

Domain‐specific zone classifications are presented in Table 3. Red classifications were uncommon across all domains (range: 0.65%–10.11%), with Emotional Health showing the highest rate (10.11%) and Resource & Resilience the lowest (0.65%). In contrast, yellow classifications were prevalent across domains, with Ownership & Activation (54.79%) and Emotional Health (52.51%) showing the highest rates. Physical Health had notably higher missing data (18.65%) compared to other domains, consistent with item‐level missingness patterns described earlier.

**TABLE 3:** Zone classification distribution by WPHS‐AV domain.

Domain		Red	Yellow	Green
Physical health	*N* (%)	644	3860	5084
		5.46	32.75	43.14
		[5.06, 5.88]	[31.90, 33.60]	[42.23, 44.03]
Emotional health	*N* (%)	1192	6189	4405
		10.11	52.51	37.37
		[9.58, 10.67]	[51.61, 53.42]	[36.50, 38.25]
Resource & resilience	*N* (%)	77	3494	8215
		0.65	29.65	69.70
		[0.52, 0.82]	[28.82, 30.48]	[68.86, 70.53]
Socioeconomics	*N* (%)	163	3630	7993
		1.38	30.80	67.82
		[1.18, 1.61]	[29.97, 31.64]	[66.96, 68.66]
Ownership & activation	*N* (%)	557	6457	4772
		4.73	54.79	40.49
		[4.35, 5.12]	[53.88, 55.69]	[39.60, 41.38]
Nutrition & lifestyle	*N* (%)	275	4595	6916
		2.33	38.99	58.68
		[2.07, 2.62]	[38.10, 39.87]	[57.78, 59.57]

*Note:* Percentage of students classified as Red (high‐need), Yellow (moderate‐need), or Green (low‐need) in each of six WPHS‐AV domains with exact 95% confidence intervals. Analyses based on first screening attempt to maximize comparability across students.

#### 3.4.2 Temporal and Repeated‐Screening Trends

Zone classifications varied significantly across screening waves. Among first screenings each academic year from Fall 2020 to Fall 2024, most domains showed improvement. Physical Health red‐zone classifications decreased from 4.7% to 1.7%, while green‐zone increased from 50% to 60% (*χ* ^2^ = 6658.6, *p* < 0.001). Emotional Health improved modestly, with green‐zone rising from 30% to 42% and red‐zone declining from 15% to 7.0% (*p* < 0.001). Ownership & Activation (green: 39%–46%; red: 6.2%–2.4%; *p* < 0.001) and Nutrition & Lifestyle (green: 50%–66%; *p* < 0.001) showed similar improvement patterns.

## 4 Discussion

High‐need (Red) classifications were uncommon across all domains, while moderate‐need (Yellow) classifications were prevalent, particularly in Emotional Health (52.51% Yellow) and Ownership & Activation (54.79% Yellow). Internal consistency across domains was acceptable for a multidomain whole‐person framework designed to identify distinct needs.

### 4.1 Temporal Trends in Zone Classification

Temporal trends in zone classifications provide preliminary descriptive evidence and cannot establish program effectiveness. The substantial decline in Emotional Health red‐zone classifications from 15% in Fall 2020 to 7.0% in Fall 2024, alongside an increase in green‐zone classifications (30%–42%), demonstrates a shift toward lower‐need classifications over the implementation period. Similar shifts occurred in Physical Health, Ownership & Activation, and Nutrition & Lifestyle. These trends may reflect pandemic recovery, program effects, or other secular changes; comparison groups would be needed to distinguish among these explanations.

### 4.2 What Whole‐Person Assessment Adds

A central contribution of WPHS‐AV is its multidomain structure, which supports identifying need profiles rather than relying on single symptom scales [33]. The tool addresses three persistent operational limitations:

Student‐linked results: Unlike anonymous surveys that produce only aggregate data, WPHS‐AV generates individualized results that enable follow‐up while maintaining appropriate confidentiality through tiered consent and data governance.

Systematic identification: Unlike reactive systems focusing on Tier 3 students with overt problems, WPHS‐AV is designed for systematic screening and identification of moderate‐need students amenable to preventive intervention, a population remaining invisible under crisis‐triggered approaches [34].

Longitudinal tracking: Administered twice annually, WPHS‐AV enables longitudinal monitoring of individual trajectories.

Table 4 compares selected whole‐person and multidomain screening instruments on key implementation dimensions. WPHS‐AV balances comprehensiveness (6 domains, 30 items) with feasibility while uniquely integrating letter‐based, color‐coded zone‐based triage (Green/Yellow/Red) and longitudinal assessment capacity designed for twice‐annual repeated administration.

**TABLE 4:** Comparison of multidomain adolescent screening tools implemented in school settings.

Instrument name	Purpose	Domains captured/measured	Intent	Year	Explicit whole‐person framework	# domains	# questions
Adolescent Health Review (AHR)	Brief multidimensional screening; risk identification; inform care plans	Psychosocial domains (e.g., family, abuse, emotional distress, substance use, sexual activity, violence)	Screening	2001	No/not explicit	14	33
Healthy Teens PDA‐based Tool	Health behavior screening; identify risks; guide counseling	Demographics, family/medical history, nutrition, activity, safety, substance use, sexuality, mental health	Screening	2009	No/not explicit	14	60–65 core (up to 90)
WPHS‐AV (Adolescent Whole Person Health Score)	Holistic whole‐person screening and assessment; identify strengths and opportunities; measure change over time	Physical Health; Emotional Health; Resource & Resilience; Socioeconomics; Ownership & Activation; Nutrition & Lifestyle	Screening + assessment	2019	Yes	6	30
SDOH Focused History Script	Facilitate SDOH conversations; support assessment and care planning	Food insecurity, housing, safety, transportation, education, social support, discrimination, access to care	Assessment	2022	No/not explicit	5	40
Secondary School Health Promotion Self‐Assessment Tool	School‐level self‐assessment; health promotion/curriculum improvement	Healthy habits, affectivity/socialization, emotional well‐being, safety/risks, specific health situations	Assessment	2022	Yes	11	18 indicators (rubric)
BEACON (Brief Evaluation of Adolescents and Children Online)	Universal mental health screening; early identification; referral	Anxiety, attention/hyperactivity, depression, eating difficulties, suicidality/self‐harm	Screening	2024	No/not explicit	4–5*	35 (grades 6–11)
Digital Health Contact (DHC)	School health/wellbeing screening; identify unmet needs; facilitate referral	Physical health, mental health, wellbeing, unmet needs (red‐flag system)	Screening	2024	No/not explicit	6	Not specified (≈20–30)

### 4.3 Developmental Appropriateness for Adolescents

The WPHS‐AV framework addresses adolescent developmental needs in ways that symptom‐focused screening cannot. Adolescence is characterized by rapid physical maturation, neurobiological changes in executive function and emotion regulation, identity exploration, and increasing autonomy from caregivers, processes that shape trajectories across health, education, and social domains [5, 35]. This is consistent with clinical staging models recognizing that early mental health difficulties may be nonspecific or transdiagnostic before developing into more clearly defined disorders [36]. The Ownership & Activation domain captures developmentally salient constructs including academic self‐efficacy, future orientation [37], and conscientiousness, reflecting adolescents' emerging capacity to influence their own outcomes. The Resource & Resilience domain assesses protective factors critical during this period, reliable adult relationships, sense of belonging, and extracurricular engagement [38], that buffer adolescents from adversity and support positive adaptation [39, 40, 41]. By integrating physical health, substance use behaviors, socioeconomic barriers, and school engagement alongside emotional health, WPHS‐AV operationalizes a whole‐person framework aligned with developmental science showing that adolescent wellbeing is interconnected across domains [42, 43]. The prevalence of needs across domains reinforces the importance of coordinated rather than siloed support systems.

### 4.4 Matching Intervention Intensity to Need Distribution

The prevalence of yellow‐zone classifications in Ownership & Activation (54.79%) and Emotional Health (52.51%) suggests that scalable preventive supports may suit many students rather than intensive clinical services. Yellow‐zone Ownership students, showing moderate concerns about academic self‐efficacy and engagement, may benefit from school counselor interventions or curriculum‐embedded programming that addresses goal‐setting and study skills. Similarly, yellow‐zone Emotional Health students may respond to peer support programs, stress‐management curricula, or structured activities that promote belonging rather than exclusively one‐on‐one behavioral health support. This distribution indicates that screening programs must invest in Tier 2 infrastructure proportional to need volume. Districts implementing screening without corresponding Tier 2 capacity risk identifying needs they cannot address, potentially undermining trust and program sustainability.

### 4.5 Implementation Lessons

Over 4 years, we encountered and addressed five major implementation challenges. These mapped primarily to the inner setting and implementation process: incomplete screening uptake, administrative bottlenecks delaying referrals, cross‐system data‐sharing barriers, consent procedures and equity tensions, and sustaining visibility for continuous quality improvement.

To improve transferability, we organize these lessons using a pragmatic lens aligned with CFIR domains: outer setting, inner setting, implementation process, and intervention characteristics. We use CFIR as an organizing framework, not a formal analytic approach, to identify factors shaping screening reach, referral timeliness, and scalability.

1. Incomplete Screening Uptake: Despite the intent to screen all eligible students, 67.7% completed the WPHS‐AV at least once. Barriers included parental consent requirements, competing classroom demands, student absences, and limited real‐time tracking. The partnership responded by extending screening windows, offering remote completion, and creating alternative identification pathways through teacher, peer, parent/caregiver, and self‐referral. This experience demonstrated that screening reach is itself an implementation outcome: universal screening depends not only on the validity of the instrument but also on administration workflows, family communication, acceptability, and timely visibility into which students have and have not been reached.
2. Administrative Bottlenecks Delaying Referrals: Initial administrative review created delays between screening and outreach. Eliminating this review step, routing screening results directly to frontline staff, and formalizing triage authority reduced median time from screening completion to first outreach from 3–4 weeks to less than 1 week. The experience also highlighted the importance of matching screening volume to response capacity: Yellow‐zone needs require scalable Tier 2 supports, while Red‐zone needs require timely clinical follow‐up. Clear workflow ownership, frontline decision‐making, and process mapping are therefore critical to translating identification into timely action.
3. Cross‐System Data‐Sharing Barriers: Coordination between schools operating under FERPA and health‐system providers operating under HIPAA required deliberate data governance infrastructure. The partnership established legally reviewed agreements defining permissible information exchange, standardized release‐of‐information procedures, signed releases before service initiation, confidentiality safeguards, and staff training. These experiences underscored that data governance should precede large‐scale screening, involve both organizational and legal stakeholders, and be reinforced through ongoing training as personnel change; written agreements alone are insufficient if frontline staff are unclear about permissible information sharing.
4. Consent Procedures and Equity Tensions: Opt‐in parental consent protected explicit family choice but also introduced potential inequities in screening access. Families facing limited English proficiency, housing instability, demanding work schedules, or administrative burden may be less likely to complete consent processes, particularly when screening authorization is embedded among numerous school‐registration forms. The partnership used multilingual materials and case‐by‐case consent for students identified outside universal screening windows, but the experience revealed an inherent tension between maximizing reach and preserving explicit family permission. Consent models should, therefore, be selected with attention to local legal requirements, community trust, family autonomy, and equity, with accessible communication and multiple opportunities for family engagement.
5. Sustaining Visibility and Continuous Quality Improvement: Limited real‐time data infrastructure initially constrained the partnership's ability to monitor school‐level completion, referral timeliness, service connection, and sustained engagement. In response, the partnership began developing automated completion dashboards and referral‐cascade tracking and instituted routine data‐review meetings with school‐site teams. This experience highlighted that implementation monitoring requires timely, actionable information available to frontline staff—not only retrospective reports to leadership. Real‐time visibility into participation, caseloads, referral volumes, and service connection can support targeted outreach, identify disparities and bottlenecks, and create feedback loops for continuous adaptation.

Detailed descriptions of these implementation barriers, adaptations, and recommendations are provided in the Appendix under “Barriers” section.

### 4.6 Program Model and Scalability

A practical contribution is not only the WPHS‐AV tool but also the operational model within which it functions. The partnership combined universal screening, integrated workflows, school‐embedded clinical capacity, and wrap‐around supports functioning as a “system of care,” including ancillary health programming and a district Wellness Center that centralize support for identified social needs. Replication will require attention to screening reach, consent, response capacity, data infrastructure, and cross‐system coordination.

### 4.7 Implications for School Health Policy, Practice, and Equity

Implementation requires formal governance, data‐sharing agreements, sustainable funding, and clearly defined staffing. California's MHSSA provided foundational funding, but implementation also required district‐county partnership, executive coordination, and iterative problem‐solving. Policies supporting school‐based screening should address governance, data exchange, and tiered response workflows, not just tool selection. Universal screening policies must be coupled with infrastructure investment in school counseling and preventive services, not only specialty behavioral health capacity.

School districts implementing whole‐person screening need three elements: student‐linked tools for individualized follow‐up, defined triage workflows for staff roles and resources, and tiered response systems matched to need distribution. The high prevalence of yellow‐zone classifications (54%–55% in Ownership & Activation and Emotional Health) indicates a need for scalable Tier 2 and preventive supports rather than intensive clinical care alone. District capacity should reflect yellow‐zone volume, not only specialty behavioral health capacity for red‐zone students.

Traditional reactive systems often overlook students with internalizing symptoms, home‐based needs, and emerging concerns. Systematic screening identified a large “missing middle” of students with moderate‐need flags, who may be less visible to crisis‐triggered systems. Bicultural and bilingual TOPSS staffing, including Parent Partners with lived experience, was designed to address access barriers for the predominantly Hispanic/Latino population.

### 4.8 Limitations

Several limitations warrant attention. First, screening reach was incomplete (67.7%) and varied across schools and waves, affecting the generalizability of prevalence estimates. Future studies should link screening rosters to district enrollment data to compare participants and nonparticipants, evaluate whether screening reach differs by school, grade, language, socioeconomic status, housing instability, disability status, or other student characteristics, and identify modifiable barriers to participation. Second, the evaluation focuses on screening classifications and triage pathways, not the full referral cascade, eligibility determination, service initiation, or clinical outcomes. Third, the WPHS‐AV lacks full psychometric validation, including construct, criterion, and test–retest reliability. Fourth, the evaluation was conducted in a single district during COVID‐19, limiting generalizability to other settings, time periods, or districts without comparable infrastructure. Fifth, although we adapted implementation in response to observed barriers, we did not formally evaluate Care Flow Plan fidelity or collect systematic student, family, or staff measures of feasibility and acceptability. Sixth, parental consent was obtained via a checkbox embedded within a multi‐program electronic registration module rather than a standalone consent process, which may have reduced visibility and contributed to incomplete screening uptake, particularly among families with limited English proficiency.

Future research should evaluate the full pathway from screening through service engagement and outcomes, examine differential attrition across student groups (by school, grade, language, socioeconomic status, housing instability, disability status, or other equity‐relevant characteristics), assess the predictive validity of WPHS‐AV classifications (subsequent service use, attendance, academic engagement, disciplinary events, and validated mental health or wellbeing outcomes), and incorporate student, family, staff, and provider perspectives on feasibility and acceptability.

## 5 Conclusion

Universal whole‐person screening, structured triage, and district‐health system partnerships can systematically identify students with moderate‐to‐high need across multiple wellbeing domains. The predominance of moderate‐need classifications highlights the importance of scalable response capacity. Successful implementation depends on screening reach, staffing, data infrastructure, and cross‐system coordination. This work demonstrates the feasibility of multidomain screening at scale in a high‐need, diverse population during unprecedented educational disruption. Future research should determine whether systematic needs identification translates into service engagement, improved outcomes, and more equitable access to support.

## Funding

This work was supported by the California Mental Health Services Oversight and Accountability Commission (MHSOAC) through California's Mental Health Student Services Act (MHSSA), authorized by Senate Bill 75 as part of the State's 2019 Budget Act. The MHSSA allocated $40 million in one‐time and $10 million in ongoing funding for school‐county mental health partnerships, funded through Proposition 63 (the Mental Health Services Act).

## Conflicts of Interest

The authors declare no conflicts of interest.

## Data Availability

The data that support the findings of this study are available from the participating unified school district and Riverside University Health System–Behavioral Health (RUHS‐BH). Restrictions apply to the availability of these data, which were used under license for this study. Data may be made available from the authors upon reasonable request and with the permission of the participating unified school district and RUHS‐BH.

## Appendix A

### Partnership Model and Governance

In 2019, the district partnered with Riverside University Health System‐Behavioral Health (RUHS‐BH) through TOPSS (Transforming Our Partnerships for Student Success), an MHSSA‐supported collaboration combining universal screening, tiered school‐based supports, and behavioral health linkage. An executive governance group met monthly to clarify partnership policies and oversee implementation. To formalize decision‐making, the group developed partnership bylaws defining roles, decision‐making authority, and dispute resolution procedures.

Each school site held structured meetings to establish and maintain working relationships between school staff and RUHS‐BH TOPSS providers. Beginning‐of‐year meetings confirmed operational procedures and aligned school leadership, student support staff, and providers on annual service plans. Regular update meetings reviewed progress, goals, and plan revisions monthly to quarterly. End‐of‐year meetings reviewed implementation data and progress toward annual goals. FERPA/HIPAA‐compliant information exchange protocols defined data sharing, timing, and parties involved.

#### Partnership Staffing and Clinical Infrastructure

School district staffing included Behavior Support Specialists, school counselors, supervised intern‐ or associate‐level therapists/social workers, Alcohol and Substance Use Intervention Specialists, and a centralized Wellness Center to coordinate support for social needs such as food, housing, and transportation. Precise district staffing counts were not consistently available across the full implementation period because school‐site staffing and collaboration patterns varied over time. In August 2022, the district launched a centralized Wellness Center to coordinate support for social needs like food, housing, and transportation.

RUHS‐BH provided TOPSS teams to support clinical follow‐up and family engagement for students requiring behavioral health services. During the study period, the RUHS‐BH TOPSS staffing complement included 1 supervisor, 1 office assistant, 3 clinical therapists, 6 Parent Partners, and 5 Transitional Age Youth Peers. These roles supported clinical assessment, therapeutic services, case management, peer support, family engagement, and coordination with school‐based teams. TOPSS teams were bicultural and bilingual, reflecting the district's predominantly Hispanic/Latino student population. Services required parental consent and Medi‐Cal eligibility screening; students ineligible for TOPSS clinical services could receive resource navigation and school‐based support.

#### Service Connection Procedures

Once a referral is accepted by the TOPSS team, the TOPSS Coordinator offers families multiple appointment options to schedule a comprehensive behavioral health assessment. During the assessment, providers confirm or screen for Medi‐Cal eligibility, as TOPSS clinical services are reimbursed through Medi‐Cal billing mechanisms. If a student does not meet Medi‐Cal eligibility criteria, the provider connects the parent/guardian to appropriate alternative resources, notifies the school of the student's ineligibility for TOPSS services, and documents outreach and follow‐up in a monthly communication log shared with school site coordinators. These students continue to receive school‐based supports for Yellow‐zone needs through district‐employed counselors and mental health staff. For students who do qualify and whose families consent to services, documentation occurs in both the health system's clinical records (under HIPAA) and the school's student information system (under FERPA, with appropriate releases). School staff document when services were initiated or declined and which specific internal and external resources were provided.

### Assessment of Potential Bias due to Missing Demographic Data

To evaluate whether incomplete demographic information biased zone assignment, we compared distributions of Red/Yellow/Green classifications between students with complete demographics (*n* = 11,016) and those with missing data (*n* = 770). A stacked chi‐square test pooling classifications across all six domains indicated a modest difference (*χ* ^2^(2) = 14.696, *p* = 0.0006). In a population‐averaged multinomial GEE model accounting for within‐student correlation across domains, demographic completeness was not associated with Red versus Green classification (OR = 1.11, 95% CI 0.92–1.36, *p* = 0.28). Students with complete demographics had modestly lower odds of Yellow versus Green classification (OR = 0.90, 95% CI 0.83–0.98, *p* = 0.020). Domain effects were significant (*p* < 0.001). Overall, missing demographic information did not appear to meaningfully affect classification into the high‐need Red zone.

### Barriers

#### Incomplete Screening Uptake (67.7%)

Despite our intent to screen all eligible students, only 67.7% completed screening at least once. Barriers included parental consent requirements, competing classroom demands, student absences, and lack of real‐time tracking. The opt‐in consent was embedded as one checkbox among approximately 20 consent statements in the electronic re‐registration module, likely reducing visibility and salience, particularly for families with limited English proficiency or those experiencing registration fatigue.

We addressed these barriers by offering multiple screening modalities, extending screening windows, and introducing alternate identification pathways. Remote links increased access for absent students, while teacher and peer referrals captured missed students during universal windows. However, the most important lesson was the need for a real‐time completion dashboard for targeted outreach.

Importantly, this incomplete reach is not a sampling limitation; it is an implementation finding. Universal screening systems must be judged not only by the validity of the screening tool, but also by whether consent procedures, administration workflows, family communication, and real‐time monitoring systems allow broad and equitable participation. This interpretation is consistent with recent systematic review evidence emphasizing that the acceptability of universal mental health screening to students, families, and school staff remains an important implementation consideration, and that understanding drivers of non‐participation is necessary to avoid widening inequities. Future implementation evaluations should therefore examine which students are not reached, why they are missed, and whether nonparticipation reflects logistical barriers, family preferences, language access, trust, or other acceptability concerns.

Based on this experience, we recommend:

1. *Investing in real‐time tracking infrastructure from day one*. Visibility into completion rates by school, grade, and student subgroup during active screening is essential for maximizing reach and identifying disparities.
2. *Weigh the trade‐offs between opt‐in and opt‐out consent*. Opt‐in respects family autonomy but creates enrollment barriers, while opt‐out maximizes participation but raises ethical concerns. Passive consent may reduce enrollment friction, especially for low‐intensity universal screening paired with opt‐in consent for services. Additionally, if opt‐in consent is required, consider standalone consent processes rather than embedding screening consent within lengthy multi‐program registration modules. Visibility and salience matter: a dedicated consent form or prominent placement may improve uptake, particularly for multilingual families navigating electronic systems.
3. *Build alternate identification pathways from the start*. Not all students will complete screening during universal administration windows, regardless of the process's design.
4. *Treat feasibility and acceptability as implementation outcomes*, not only post hoc evaluation questions. Screening reach should be monitored in real time, and outreach strategies should be adapted based on completion patterns, family feedback, language needs, and school‐site workflow barriers. In practice, this requires routine review of completion data, referral volumes, and service‐connection rates with frontline staff so that implementation can be adjusted before participation gaps become entrenched.

#### Administrative Bottlenecks Delaying Referrals

We implemented three changes to address the bottleneck: eliminating the administrative review step, establishing direct routing from the screening platform to frontline staff, and formalizing triage decision‐making authority with frontline staff. The impact was significant. Median time from screening completion to first outreach attempt reduced from 3 to 4 weeks to less than 1 week.

This bottleneck also highlighted the importance of matching screening volume to available response capacity. The program did not include a formal staffing‐capacity analysis, and staffing levels varied across sites and over time. However, the response model relied on a layered infrastructure: Yellow‐zone needs were routed primarily to district‐employed school counselors, Behavior Support Specialists, school‐based mental health staff, Alcohol and Substance Use Intervention Specialists, and the Wellness Center, while Red‐zone needs were routed to TOPSS teams for clinical follow‐up. The improvement in time to first outreach after direct routing suggests that workflow design, not only staffing volume, affected responsiveness. At the same time, the high prevalence of Yellow classifications underscores that universal screening cannot be implemented as a measurement activity alone; it requires explicit planning for Tier 2 staffing, group‐based supports, referral management, and sustainable funding proportional to the number of students likely to screen positive for moderate need.

Staff ownership of the triage process increased, as counselors and TOPSS coordinators reported greater clarity about their roles and authority. Student and family engagement improved, likely because timely follow‐up signals the school's seriousness about screening.

Three key lessons emerged: process mapping before implementation would have identified the bottleneck, frontline staff should own triage decisions due to their clinical expertise and direct relationships with students, and speed matters in translating screening results into action to prevent erosion of trust and undermine the value of systematic needs identification.

#### Cross‐System Data Sharing Barriers

School districts operate under FERPA, while health systems operate under HIPAA. Sharing student information across these frameworks required navigating complex legal requirements. We addressed legal review of data‐sharing agreements, defined minimum necessary information exchange, trained staff on information sharing, and developed student‐specific releases for coordination between school staff and TOPSS providers.

We implemented a multi‐component solution for compliant data governance: (1) formal agreements reviewed by legal counsel specifying data elements, personnel, and sharing purposes; (2) standardized release‐of‐information forms for school‐TOPSS coordination; (3) signed releases before service initiation, acknowledging families' consent to limited information exchange; and (4) operational confidentiality procedures coding therapy absences as ‘School Business’ to protect privacy.

Four lessons emerged: data governance must precede screening; establishing compliant data‐sharing infrastructure involves multiple stakeholders; reviewing templates from other school–health system partnerships saves time and reduces errors; and ongoing training is crucial as staff turnover occurs, as written agreements alone are insufficient if frontline personnel lack understanding of information sharing and confidentiality.

#### Consent Procedures and Equity Tensions

Our opt‐in consent model, requiring parental permission before students could complete screening, created potential equity gaps that concerned us. Families with limited English proficiency might not fully understand consent forms even when translated. Families experiencing housing instability might not receive enrollment packets delivered via mail or sent home with students. Busy working parents, particularly those juggling multiple jobs or shift work, might overlook permission slips among numerous back‐to‐school forms. Yet shifting to passive opt‐out consent raised ethical concerns about screening students without explicit parental permission, particularly for assessments covering sensitive topics such as emotional health, substance use, and family socioeconomic circumstances.

We implemented a compromise approach that prioritized explicit consent while attempting to reduce barriers. We used opt‐in consent requiring parental permission slips included in enrollment packets. We allowed case‐by‐case consent for students referred by teachers or peers outside the universal screening windows, enabling individualized outreach to families when school staff identified specific concerns. We provided consent materials in multiple languages reflecting our district's linguistic diversity, particularly Spanish given the 65% Hispanic/Latino student population.

However, we recognize this approach likely contributed to our 67.7% completion rate and may have excluded students whose families face the greatest barriers to navigating school administrative processes. We offer several recommendations for other implementers navigating this tension. First, carefully weigh community values, legal requirements, and equity implications when selecting between opt‐in and opt‐out consent models; there is no universal right answer, and the appropriate choice depends on local context, community trust levels, and regulatory environment. Second, if using opt‐in consent, maximize touchpoints beyond enrollment packets—consider back‐to‐school nights, parent–teacher conferences, phone calls home, and text message reminders, and provide multilingual materials and community education about screening purposes and how results will be used. Third, if using opt‐out consent, invest in robust community engagement beforehand to build understanding and trust, establish clear and accessible opt‐out procedures, and ensure culturally responsive communication that respects family autonomy. Fourth, consider hybrid models such as opt‐out consent for initial screening paired with opt‐in consent before providing services, balancing broad needs identification with explicit permission before intervention.

#### Sustaining Visibility and Continuous Quality Improvement

Without real‐time data infrastructure, we struggled to monitor implementation quality and identify improvement opportunities. We could not readily determine which schools had high or low completion rates, whether students with Red flags actually connected to TOPSS services, how much time elapsed from referral to first appointment, or whether students remained engaged with services over time. This limited our ability to identify disparities, troubleshoot barriers, and demonstrate value to stakeholders.

We are addressing this through three ongoing infrastructure investments. First, we are developing an automated dashboard displaying completion rates by school site, grade level, and screening wave, enabling rapid identification of participation gaps and targeted outreach to underperforming sites. Second, we are building referral cascade tracking that follows students from screening through triage to service connection and engagement, quantifying what proportion of Yellow and Red classifications result in actual service delivery and identifying where students fall out of the pathway. Third, we have instituted quarterly data review meetings with school site teams to examine site‐specific implementation data, identify barriers, and collaboratively problem‐solve adaptations.

This experience taught us three critical lessons about implementation monitoring. First, effective monitoring requires dedicated data infrastructure rather than periodic manual reports; staff cannot act on information they receive weeks or months after implementation challenges emerge. Second, dashboards must be accessible to frontline staff, not only executives, to drive action; counselors and TOPSS coordinators need real‐time visibility into their caseloads, referral volumes, and service connection rates to manage workflow and prioritize outreach. Third, feedback loops enable continuous improvement; without systematic data review, partnerships rely on anecdotal problem identification rather than proactive monitoring and adaptation.

## References

[1] L. A. Lebrun‐Harris , R. M. Ghandour , M. D. Kogan , and M. D. Warren , “Five‐Year Trends in US Children's Health and Well‐Being, 2016‐2020,” JAMA Pediatrics 176, no. 7 (2022): e220056, 10.1001/jamapediatrics.2022.0056. PMC8922203 35285883

[2] R. H. Bitsko , J. R. Holbrook , R. M. Ghandour , et al., “Epidemiology and Impact of Health Care Provider–Diagnosed Anxiety and Depression Among US Children,” Journal of Developmental & Behavioral Pediatrics 39, no. 5 (2018): 395–403, 10.1097/DBP.0000000000000571. PMC6003874 29688990

[3] R. C. Kessler , P. Berglund , O. Demler , R. Jin , K. R. Merikangas , and E. E. Walters , “Lifetime Prevalence and Age‐Of‐Onset Distributions of DSM‐IV Disorders in the National Comorbidity Survey Replication,” Archives of General Psychiatry 62, no. 6 (2005): 593–602, 10.1001/archpsyc.62.6.593. 15939837

[4] J. J. Arnett , “Emerging Adulthood: What Is It, and What Is It Good for?,” Child Development Perspectives 1, no. 2 (2007): 68–73, 10.1111/j.1750-8606.2007.00016.x.

[5] J. Piaget , “Part I: Cognitive Development in Children: Piaget Development and Learning,” Journal of Research in Science Teaching 2, no. 3 (1964): 176–186, 10.1002/tea.3660020306.

[6] S. E. Jones , “Mental Health, Suicidality, and Connectedness Among High School Students During the COVID‐19 Pandemic—Adolescent Behaviors and Experiences Survey, United States, January–June 2021,” MMWR Supplement 71 (2022): su7103a3, 10.15585/mmwr.su7103a3. PMC8979602 35358165

[7] M. R. Kaufman , K. Wright , J. Simon , G. Edwards , J. Thrul , and D. L. DuBois , “Mentoring in the Time of COVID‐19: An Analysis of Online Focus Groups With Mentors to Youth,” American Journal of Community Psychology 69, no. 1–2 (2022): 33–45, 10.1002/ajcp.12543. PMC8426756 34318506

[8] X. Zhang , S. Tennyson , C. L. Kjolhede , and W. M. Brunner , “School‐Based Health Centers and the Utilization of Primary Care in Rural Communities,” American Journal of Preventive Medicine 69, no. 4 (2025): 107962, 10.1016/j.amepre.2025.107962. PMC12389262 40588046

[9] M. Reinert , D. Fritze , and T. Nguyen , The State of Mental Health in America (2021).

[10] Unpacking California's Chronic Absence Crisis Through 2023–24 | Policy Analysis for California Education (2025), accessed January 21, 2026, https://edpolicyinca.org/publications/unpacking‐californias‐chronic‐absence‐crisis‐through‐2023‐24.

[11] Mental Health Services Act – CalEdFacts (CA Dept of Education) accessed January 21, 2026, https://www.cde.ca.gov/ls/mh/cefmentalhealth.asp.

[12] C. J. Heinrich , A. Colomer , and M. Hieronimus , “Minding the Gap: Evidence, Implementation and Funding Gaps in Mental Health Services Delivery for School‐Aged Children,” Children and Youth Services Review 150 (2023): 107023, 10.1016/j.childyouth.2023.107023. PMC10202463 37261333

[13] S. Hossain , S. O'Neill , and I. Strnadová , “What Constitutes Student Well‐Being: A Scoping Review of Students' Perspectives,” Child Indicators Research 16, no. 2 (2023): 447–483, 10.1007/s12187-022-09990-w. PMC9668225 36405573

[14] K. L. Lane , W. P. Oakes , R. P. Ennis , and S. E. Hirsch , “Identifying Students for Secondary and Tertiary Prevention Efforts: How Do We Determine Which Students Have Tier 2 and Tier 3 Needs?,” Preventing School Failure: Alternative Education for Children and Youth 58, no. 3 (2014): 171–182, 10.1080/1045988X.2014.895573.

[15] Teacher Recognition, Concern, and Referral of Children's Internalizing and Externalizing Behavior Problems (Office of Justice Programs, 2026), accessed January 21, 2026, https://www.ojp.gov/library/publications/teacher‐recognition‐concern‐and‐referral‐childrens‐internalizing‐and.

[16] E. H. Connors , K. Moffa , T. Carter , et al., “Advancing Mental Health Screening in Schools: Innovative, Field‐Tested Practices and Observed Trends During a 15‐Month Learning Collaborative,” Psychology in the Schools 59, no. 6 (2022): 1135–1157, 10.1002/pits.22670. PMC9307132 35875829

[17] V. Keeton , S. Soleimanpour , and C. D. Brindis , “School‐Based Health Centers in an Era of Health Care Reform: Building on History,” Current Problems in Pediatric and Adolescent Health Care 42, no. 6 (2012): 132–158, 10.1016/j.cppeds.2012.03.002. PMC3770486 22677513

[18] J. Dryfoos and S. Maguire , Inside Full‐Service Community Schools (Corwin Press, 2002).

[19] F. Rowe , D. Stewart , and C. Patterson , “Promoting School Connectedness Through Whole School Approaches,” Health Education 107, no. 6 (2007): 524–542, 10.1108/09654280710827920.

[20] School‐based health centers | County Health Rankings & Roadmaps (2024), accessed January 21, 2026, https://www.countyhealthrankings.org/strategies‐and‐solutions/what‐works‐for‐health/strategies/school‐based‐health‐centers.

[21] School‐Based Health Centers—School‐Based Health Alliance (2026), accessed January 21, 2026, https://www.sbh4all.org/school‐based‐health‐centers/.

[22] School‐Based Health Centers and Behavioral Health Care for Students Enrolled in Medicaid (MACPAC, 2025), accessed January 21, 2026, https://www.macpac.gov/publication/school‐based‐health‐centers‐and‐behavioral‐health‐care‐for‐students‐enrolled‐in‐medicaid/.

[23] D. Khurana , G. Leung , B. Sasaninia , D. Tran , M. Khan , and A. Firek , “The Whole PERSON Health Score: A Patient‐Focused Tool to Measure Nonmedical Determinants of Health,” NEJM Catalyst 3, no. 8 (2022): CAT.22.0096, 10.1056/CAT.22.0096.

[24] Selected Statistics on Enrollment, Staff, and Graduates in Public School Districts Enrolling More Than 15,000 Students in Fall 2021: Selected Years, 1990 Through 2021 (National Center for Education Statistics, Institute of Education Sciences, U.S. Department of Education, 2024), accessed August 13, 2026, https://nces.ed.gov/programs/digest/d22/tables/dt22_215.10.asp.

[25] Agency TE , Multi‐tiered System of Supports (MTSS) (2024), accessed January 21, 2026, https://tea.texas.gov/academics/special‐student‐populations/special‐education/programs‐and‐services/multi‐tiered‐system‐of‐supports‐mtss.

[26] Multi‐Tiered System of Supports | Department of Education (2026), accessed January 21, 2026, https://educate.iowa.gov/pk‐12/student‐supports/integrated‐supports/mtss.

[27] R. F. DeVellis and C. T. Thorpe , Scale Development: Theory and Applications, fifth ed. (Sage, 2022).

[28] G. O. Boateng , T. B. Neilands , E. A. Frongillo , H. R. Melgar‐Quiñonez , and S. L. Young , “Best Practices for Developing and Validating Scales for Health, Social, and Behavioral Research: A Primer,” Frontiers in Public Health 6 (2018): 149, 10.3389/fpubh.2018.00149. PMC6004510 29942800

[29] T. R. Hinkin , “A Brief Tutorial on the Development of Measures for Use in Survey Questionnaires,” Organizational Research Methods 1, no. 1 (1998): 104–121, 10.1177/109442819800100106.

[30] C. J. Vorland , M. M. Bohan Brown , M. I. Cardel , and A. W. Brown , “Traffic Light Diets for Childhood Obesity: Disambiguation of Terms and Critical Review of Application, Food Categorization, and Strength of Evidence,” Current Developments in Nutrition 6, no. 3 (2022): nzac006, 10.1093/cdn/nzac006. PMC8929981 35317412

[31] D. Khurana , M. Garner , B. Bass , B. Sasininia , G. Leung , and A. Firek , “Sexual‐Orientation Disparities in Whole Person Health: Age‐ and Gender‐Stratified Analysis of Patients in a Safety‐Net System,” International Journal for Equity in Health 24, no. 1 (2025): 310, 10.1186/s12939-025-02672-3. PMC12617001 41233865

[32] D. Khurana and A. Short , “Protecting Safety‐Net Equity Monitoring After Federal SOGI Data Rollbacks,” Health Affairs Scholar 4 (2026): qxag124, 10.1093/haschl/qxag124. PMC13235683 42253878

[33] K. Göbel and C. Cohrdes , “The Whole Is Greater Than the Sum of Its Parts: Profiles of Multiple Mental Health Risk Factors Using Latent Class Analysis,” Child and Adolescent Psychiatry and Mental Health 15 (2021): 27, 10.1186/s13034-021-00380-8. PMC8204434 34127038

[34] Center LDP , House Co‐Sponsorship Memo 43670 Information; 2025‐2026 Regular Session—PA House of Representatives (2026), accessed January 21, 2026, https://www.palegis.us/house/co‐sponsorship/memo.

[35] J. A. Knopf , R. K. C. Finnie , Y. Peng , et al., “School‐Based Health Centers to Advance Health Equity,” American Journal of Preventive Medicine 51, no. 1 (2016): 114–126, 10.1016/j.amepre.2016.01.009. PMC5759331 27320215

[36] P. D. McGorry , C. Mei , N. Dalal , et al., “The Lancet Psychiatry Commission on Youth Mental Health,” Lancet Psychiatry 11, no. 9 (2024): 731–774, 10.1016/S2215-0366(24)00163-9. 39147461

[37] S. Lindstrom Johnson , R. W. Blum , and T. L. Cheng , “Future Orientation: A Construct With Implications for Adolescent Health and Wellbeing,” International Journal of Adolescent Medicine and Health 26, no. 4 (2014): 459–468, 10.1515/ijamh-2013-0333. PMC4827712 24523304

[38] N. A. Ruvalcaba , J. Gallegos , A. Borges , and N. Gonzalez , “Extracurricular Activities and Group Belonging as a Protective Factor in Adolescence,” Psicología Educativa 23, no. 1 (2017): 45–51, 10.1016/j.pse.2016.09.001.

[39] M. Ungar , “Resilience, Trauma, Context, and Culture,” Trauma, Violence & Abuse 14, no. 3 (2013): 255–266, 10.1177/1524838013487805. 23645297

[40] A. S. Masten , C. M. Lucke , K. M. Nelson , and I. C. Stallworthy , “Resilience in Development and Psychopathology: Multisystem Perspectives,” Annual Review of Clinical Psychology 17, no. 1 (2021): 521–549, 10.1146/annurev-clinpsy-081219-120307. 33534615

[41] M. O. Wright and A. S. Masten , “Resilience Processes in Development,” in Handbook of Resilience in Children, ed. S. Goldstein and R. B. Brooks (Springer US, 2005), 17–37, 10.1007/0-306-48572-9_2.

[42] I. I. McKinnon , K. H. Krause , L. Robin , et al., “Experiences of Unstable Housing Among High School Students—Youth Risk Behavior Survey, United States, 2021,” MMWR Supplements 72, no. 1 (2023): 29–36, 10.15585/mmwr.su7201a4. PMC10156156 37104394

[43] K. S. Cain , S. C. Meyer , E. Cummer , et al., “Association of Food Insecurity With Mental Health Outcomes in Parents and Children: A Systematic Review,” Academic Pediatrics 22, no. 7 (2022): 1105–1114, 10.1016/j.acap.2022.04.010. PMC10153634 35577282

